# Toward Precision Oral Medicine in Pemphigus Vulgaris: A Conceptual AI Framework for Rituximab Response Prediction

**DOI:** 10.3390/ph19081292

**Published:** 2026-08-15

**Authors:** Emily-Alice Russu, Radu Ilinca, Liliana Gabriela Popa, Anca Silvia Dumitriu, Stana Păunică, Călin Giurcăneanu, Cristina-Crenguța Albu

**Affiliations:** 1 Doctoral School, “Carol Davila” University of Medicine and Pharmacy, 020021 Bucharest, Romania; emily-alice.russu@drd.umfcd.ro; 2 Department of Medical Informatics and Biostatistics, Faculty of Dentistry, “Carol Davila” University of Medicine and Pharmacy, 020021 Bucharest, Romania; radu.ilinca@umfcd.ro; 3 Department of Oncologic Dermatology, Elias Emergency University Hospital, “Carol Davila” University of Medicine and Pharmacy, 020021 Bucharest, Romania; liliana.popa@umfcd.ro (L.G.P.); calin.giurcaneanu@umfcd.ro (C.G.); 4 Department of Periodontology, Faculty of Dentistry, “Carol Davila” University of Medicine and Pharmacy, 020021 Bucharest, Romania; 5 Department of Genetics, Faculty of Dentistry, “Carol Davila” University of Medicine and Pharmacy, 020021 Bucharest, Romania; cristina.albu@umfcd.ro

**Keywords:** pemphigus vulgaris, rituximab, pharmacogenomics, artificial intelligence, machine learning, biomarkers, oral phenotype, precision oral medicine, electronic health records

## Abstract

**Background/Objectives:** Rituximab has improved outcomes in pemphigus vulgaris (PV), but treatment response remains heterogeneous and no clinically validated biomarker currently supports reliable individual-level prediction. Oral lesions frequently represent the earliest manifestation of PV and contribute substantially to disease burden and longitudinal assessment. This perspective aimed to synthesize candidate biomarkers and organize them within a conceptual, task-specific artificial intelligence (AI) framework for future rituximab outcome modeling. **Methods:** A structured literature search and narrative synthesis evaluated genetic and pharmacogenomic, immunological and serological, cellular, clinical, and oral phenotypic variables potentially relevant to rituximab response. Variables were considered according to biological rationale, available evidence, temporal availability, and potential use in pretreatment prognosis or post-treatment response monitoring. No patient-level dataset was analyzed, and no model was trained or validated. **Results:** Candidate pharmacogenomic variables include *FCGR3A* rs396991, *FCGR2A* rs1801274, and *IL6* rs1800795, although evidence derives mainly from autoimmune diseases other than PV. *IL10*, *TNF*, *TNFRSF13B*, and population-specific HLA variables remain exploratory. Anti-desmoglein autoantibodies, BAFF, cytokine profiles, CD19^+^ B-cell depletion, CD27^+^ memory B-cell repopulation, regulatory T-cell dynamics, natural killer cell phenotypes, and clinical and oral phenotypic variables may provide complementary information, but none currently demonstrates sufficient independent predictive validity. The proposed RTX-AI Response Framework distinguishes the Pretreatment Prediction Component from the Post-Treatment Response Monitoring Component and assigns no numerical weights, probabilities, thresholds, or treatment recommendations. **Conclusions:** The framework should be interpreted exclusively as a conceptual research architecture. Prospective multicenter data collection, standardized biomarker assessment, task-specific model development, calibration, external validation, and evaluation of clinical utility are required before potential clinical implementation.

## 1. Introduction

Pemphigus vulgaris (PV) is a rare autoimmune blistering disease characterized by the development of mucocutaneous erosions resulting from the loss of intercellular adhesion between keratinocytes. The pathogenic process is primarily mediated by immunoglobulin G (IgG) autoantibodies directed against desmoglein 3 (DSG3) and desmoglein 1 (DSG1), two essential desmosomal cadherins involved in maintaining epithelial integrity [1,2]. Although PV is considered an uncommon disorder, it is associated with substantial morbidity, impaired quality of life, and, in the absence of adequate treatment, potentially life-threatening complications [3].

Oral lesions frequently represent the earliest clinical manifestation of PV and may precede cutaneous involvement by several months. In some patients, oral lesions remain the sole manifestation of the disease for prolonged periods, emphasizing the pivotal role of oral healthcare professionals in the early recognition, diagnosis, and long-term monitoring of affected individuals [4]. Painful erosions, desquamative gingivitis, dysphagia, impaired nutritional intake, and speech difficulties may severely compromise oral function and overall well-being, underscoring the importance of integrating oral medicine into the multidisciplinary management of PV [4,5,6].

For decades, systemic corticosteroids and conventional immunosuppressive agents represented the cornerstone of PV treatment [7]. However, the introduction of rituximab, a chimeric anti-CD20 monoclonal antibody, has fundamentally transformed the therapeutic landscape of this disease. Rituximab induces B-cell depletion through multiple mechanisms, including antibody-dependent cellular cytotoxicity, complement-dependent cytotoxicity, and apoptosis induction [8]. Several clinical studies have demonstrated its superiority over conventional therapeutic approaches in achieving sustained remission while reducing cumulative corticosteroid exposure and treatment-related adverse effects [7,9,10].

Despite these therapeutic advances, treatment outcomes remain highly heterogeneous. While some patients achieve prolonged complete remission following a single treatment cycle, others experience early relapse or exhibit an inadequate clinical response [11,12]. This variability suggests the existence of individual biological determinants influencing treatment efficacy and highlights the need for reliable predictive biomarkers capable of supporting therapeutic decision-making and optimizing targeted interventions [13].

Recent advances in genomic sequencing technologies, multi-omics analytical platforms, and high-throughput immunological profiling have facilitated the identification of an increasing number of biomarkers potentially associated with response to biological therapies. Genetic variants involved in immune regulation and rituximab pharmacodynamics, serological markers reflecting disease activity, and cellular biomarkers associated with immune reconstitution have all emerged as promising candidates [11,13,14]. Nevertheless, most studies have evaluated these biomarkers independently, without integrating information derived from multiple biological domains into a unified predictive framework.

In parallel, artificial intelligence (AI) and machine learning (ML) have emerged as powerful tools for analyzing complex biomedical datasets and identifying clinically relevant patterns that may not be detectable through conventional statistical approaches [15,16]. By integrating genetic, immunological, cellular, clinical, and oral phenotypic information within Electronic Health Record (EHR)-based systems, AI-driven predictive models may facilitate individualized estimates of the probability of response to rituximab and support the implementation of precision medicine strategies in patients with PV [17,18]. Moreover, the incorporation of oral phenotypic characteristics, including lesion severity, distribution, and the presence of desquamative gingivitis, may provide additional predictive value and reinforce the contribution of oral health professionals to multidisciplinary care pathways.

This perspective review critically examines the genetic, immunological, cellular, and clinical biomarkers associated with rituximab response in PV and explores the potential application of artificial intelligence in the development of predictive platforms for precision oral medicine. Furthermore, a conceptual framework integrating multimodal biomarkers through AI-based approaches is proposed, together with the RTX-AI Response Framework, which is intended to support future prospective model-development and validation studies.

From a pharmaceutical perspective, optimizing biological interventions in PV remains a major unmet clinical need. Although rituximab has substantially improved disease outcomes, considerable interindividual variability in therapeutic response persists [7,9,10,11,12,13]. The identification of pharmacogenomic and immunological biomarkers capable of predicting treatment efficacy may facilitate patient stratification, improve the selection of targeted therapies, reduce unnecessary drug exposure, and support the future development of precision oral medicine approaches [13,14,15]. Artificial intelligence-based approaches may further enhance these efforts by integrating multimodal datasets into clinically meaningful predictive frameworks [14,15,16,17].

### Literature Search Strategy and Evidence Selection

A structured literature search was conducted to identify publications relevant to pemphigus vulgaris, rituximab response, candidate predictive and monitoring biomarkers, pharmacogenomics, artificial intelligence, machine learning, Electronic Health Records, and precision medicine. PubMed/MEDLINE, Scopus, and Web of Science Core Collection were searched from database inception to 18 May 2026. Targeted supplementary searches and forward citation checking were subsequently conducted through 5 August 2026 to identify newly published studies relevant to the revised manuscript.

The search strategy combined controlled vocabulary, where available, with free-text terms related to the disease, intervention, biomarkers, and computational framework. Core search concepts included: “pemphigus vulgaris”, “pemphigus”, “rituximab”, “anti-CD20”, “treatment response”, “remission”, “relapse”, “biomarker”, “predictor”, “prognostic marker”, “pharmacogenomics”, “*FCGR3A*”, “*FCGR2A*”, “BAFF”, “BLyS”, “desmoglein 1”, “desmoglein 3”, “anti-DSG1”, “anti-DSG3”, “CD19”, “CD27”, “B-cell depletion”, “B-cell repopulation”, “cytokine”, “HLA”, “artificial intelligence”, “machine learning”, “Electronic Health Record”, “Digital Twin”, and “precision medicine”. Boolean operators were adapted to the syntax of each database. The complete search strategy is provided in Supplementary Table S1.

Publications were considered for inclusion when they addressed at least one of the following domains: (i) clinical response, remission, relapse, or treatment failure following rituximab therapy in pemphigus vulgaris; (ii) genetic, pharmacogenomic, serological, immunological, cellular, clinical, or oral phenotypic biomarkers potentially associated with rituximab response or disease outcome; (iii) artificial intelligence, machine-learning, EHR, explainable AI, or Digital Twin approaches relevant to predictive modeling or precision medicine; or (iv) methodological principles applicable to the development, validation, calibration, implementation, or continuous updating of clinical prediction models.

Priority was given to systematic reviews, meta-analyses, randomized or prospective clinical studies, observational cohorts, translational biomarker studies, consensus documents, methodological guidance, and landmark publications. Case reports, narrative reviews, and studies conducted in related autoimmune or inflammatory diseases were considered only when they provided mechanistic, methodological, or technological information not yet available specifically for pemphigus vulgaris.

Publications were excluded when they were not relevant to rituximab response, biomarker evaluation, artificial intelligence-based prediction, or precision medicine; when they lacked sufficient methodological or clinical detail; or when they were available only as conference abstracts without an accessible full-text report. No formal risk-of-bias assessment or quantitative evidence synthesis was performed because this article was designed as a perspective rather than a systematic review.

The reference lists of included articles and relevant reviews were manually screened to identify additional eligible publications. Forward citation searching was also performed for selected landmark studies when necessary to identify subsequent validation or methodological publications. The evidence was narratively synthesized according to biomarker domain, temporal availability relative to rituximab initiation, and potential role in pretreatment prediction or post-treatment monitoring.

## 2. From Individual Biomarkers to Artificial Intelligence-Driven Precision Oral Medicine

### 2.1. Oral Manifestations of Pemphigus Vulgaris: From Diagnostic Clues to Predictive Biomarkers

Oral manifestations represent one of the most clinically relevant features of PV and frequently constitute the earliest expression of the disease. Oral mucosal lesions may precede cutaneous involvement and, in some patients, may remain the sole clinical manifestation for prolonged periods. However, the reported frequency of initial or isolated oral involvement varies across studies because of differences in study populations, clinical definitions, and methods of assessment [19,20]. Consequently, dental practitioners are often uniquely positioned to recognize the disease at an early stage and initiate appropriate diagnostic investigations.

From a pathogenic perspective, oral involvement is largely explained by the predominant expression of desmoglein 3 within the oral epithelium [21]. IgG autoantibodies directed against this desmosomal cadherin induce acantholysis and disrupt intercellular adhesion between keratinocytes, resulting in the formation of fragile blisters that rapidly rupture and evolve into persistent, painful erosions [1,2].

The most frequently affected sites include the buccal mucosa, soft palate, attached gingiva, ventral surface of the tongue, lips, and floor of the mouth [4,5,19,20,21]. Clinically, patients commonly experience severe oral pain, impaired food intake, dysphagia, speech difficulties, and a substantial reduction in quality of life [6].

Desquamative gingivitis deserves particular attention within dental practice, as it may represent the only clinically detectable sign during the initial stages of the disease [21]. Under these circumstances, distinguishing PV from other immune-mediated disorders affecting the oral mucosa, such as mucous membrane pemphigoid or erosive oral lichen planus, is of paramount importance [5,22].

Within the emerging framework of precision oral medicine, the oral phenotype may provide valuable prognostic and predictive information. Variables such as the severity of oral involvement, the number of affected anatomical sites, the presence of desquamative gingivitis, the persistence of erosive lesions, and the response of oral manifestations to treatment may complement genetic, immunological, and cellular biomarkers in the development of integrated predictive models [12,13].

Furthermore, advances in digital imaging technologies and automated image analysis algorithms open new avenues for the objective monitoring of oral lesions. Standardized intraoral photographs analyzed through artificial intelligence-based approaches may facilitate disease severity quantification and early relapse detection, thereby supporting individualized therapeutic strategies [23,24].

The integration of oral clinical data into EHR systems and Digital Twin models may ultimately transform oral manifestations from simple diagnostic indicators into digital biomarkers with predictive value for biological treatment outcomes [25,26]. Within the proposed AI platform, these features are collectively incorporated under the concept of the oral phenotype, encompassing lesion severity, anatomical distribution, the presence of desquamative gingivitis, and the functional burden imposed by the disease.

### 2.2. Rituximab as a Targeted Drug Intervention in Pemphigus Vulgaris

Rituximab, a chimeric anti-CD20 monoclonal antibody, has emerged as a cornerstone in the management of PV. Through antibody-dependent cellular cytotoxicity, complement-dependent cytotoxicity, and apoptosis induction, rituximab effectively depletes CD20-positive B cells involved in autoantibody production [8]. Clinical trials have demonstrated superior efficacy compared with conventional immunosuppressive regimens, resulting in higher remission rates and reduced cumulative corticosteroid exposure [7,9,10]. Consequently, rituximab is currently recommended as a first-line treatment option for moderate-to-severe PV [7,27].

Despite these advances, therapeutic outcomes remain heterogeneous. While some patients achieve prolonged remission following a single treatment cycle, others experience early relapse or incomplete responses [11,12]. Several factors may contribute to this variability, including disease duration, baseline immunological status, concomitant therapies, and genetic determinants influencing rituximab pharmacodynamics [13,28].

Rituximab exposure is influenced not only by patient-related biological factors but also by the administered induction regimen. Regimens used in pemphigus include the rheumatoid arthritis protocol, generally consisting of two 1000 mg infusions administered 2 weeks apart; the lymphoma-derived protocol of 375 mg/m^2^ weekly for four consecutive weeks; and several immunologically guided or fixed low-dose approaches. Although comparative studies have not established a universally optimal regimen for all patients with PV, differences in cumulative dose, infusion schedule, concomitant corticosteroid exposure, disease burden, and retreatment strategy may affect the depth and duration of B-cell depletion and may therefore confound associations between candidate biomarkers and clinical outcomes. Future pharmacogenomic and AI-based studies should consequently record the exact rituximab regimen, cumulative administered dose, body-surface-area-adjusted exposure, concomitant immunosuppression, retreatment schedule, and treatment adherence as mandatory covariates rather than attributing outcome variability primarily to genetic factors [29,30,31].

Direct pharmacological markers may also provide clinically relevant information. Serum rituximab concentrations, pharmacokinetic exposure, and the development of anti-rituximab antibodies, including human anti-chimeric antibodies, have been associated with altered drug clearance, incomplete B-cell depletion, and reduced or lost therapeutic response in several rituximab-treated immune-mediated diseases [32,33,34]. Evidence in PV remains limited, and standardized thresholds have not been established; nevertheless, serum drug levels and anti-drug antibody status should be considered candidate post-treatment pharmacological variables in future prospective studies, particularly in patients with incomplete depletion or secondary non-response. Because these variables become available only after treatment initiation, they would belong exclusively to the Post-Treatment Response Monitoring Component [32,33,34].

The biological effect of rituximab may additionally depend on target and effector-pathway characteristics beyond single-nucleotide variants. Potential research variables include the density of CD20 expression on circulating or tissue B-cell subsets, genetic or transcript-level variation in *MS4A1* encoding CD20, *FCGR3A* copy-number variation, natural-killer-cell abundance and functional state, and the relative contribution of antibody-dependent cellular cytotoxicity, antibody-dependent cellular phagocytosis, and complement-dependent cytotoxicity. These factors remain insufficiently studied in PV and should be regarded as mechanistic candidates rather than established predictors [8,35,36].

The identification of biomarkers associated with treatment efficacy may therefore facilitate the optimization of rituximab intervention strategies. Pharmacogenomic profiling, together with serological and cellular biomarkers, may contribute to individualized treatment selection, support decisions regarding retreatment timing, and minimize unnecessary exposure to costly biological therapies [11,12,13]. Such approaches align with the principles of precision medicine and represent a promising direction for future drug intervention strategies in oral health-related diseases [14,15].

Rituximab should also be considered within an evolving therapeutic landscape rather than as the only targeted intervention under investigation for PV. Emerging approaches include inhibition of the neonatal Fc receptor with efgartigimod, Bruton tyrosine kinase inhibition with rilzabrutinib, and antigen-specific cellular strategies such as desmoglein 3-directed chimeric autoantibody receptor T cells [37,38,39]. These therapies act through mechanisms that differ substantially from CD20-mediated B-cell depletion and may require distinct predictive and monitoring biomarkers. Nevertheless, the task-specific principles proposed in the present framework—including explicit index times, temporally eligible predictors, harmonized outcomes, calibration, external validation, and assessment of clinical utility—could be adapted to future outcome models developed for these agents. Agent-specific frameworks would still be required, and biomarkers proposed for rituximab should not be assumed to generalize automatically to other targeted therapies [39,40].

Several genetic variants have been investigated as potential modifiers of rituximab response in autoimmune diseases, although direct evidence in pemphigus vulgaris remains extremely limited [28,41]. Accordingly, the variants summarized in Table 1 are presented as mechanistically plausible candidates for future PV-specific evaluation rather than as validated pharmacogenomic biomarkers. The principal candidate variants and their biological rationale are summarized in Table 1.

### 2.3. Candidate Pharmacogenomic Variants and Their Mechanistic Relationship to Rituximab Response

Rituximab is a chimeric immunoglobulin G1 monoclonal antibody directed against CD20 expressed on pre-B and mature B lymphocytes. Its therapeutic activity involves antibody-dependent cellular cytotoxicity, complement-dependent cytotoxicity, antibody-dependent cellular phagocytosis, and direct induction of cell death. Genetic variants affecting Fcγ-receptor affinity, inflammatory cytokine production, and B-cell survival signaling may therefore represent biologically plausible modifiers of rituximab pharmacodynamics [8,28,41].

Among the candidate Fcγ-receptor variants, *FCGR3A* rs396991 is mechanistically plausible but should not be presented as an established predictor of rituximab response in PV. This nonsynonymous variant produces a phenylalanine-to-valine substitution at amino acid position 158, commonly described as *FCGR3A* F158V or V158F. The 158V allotype has higher affinity for the Fc portion of immunoglobulin G1 and may enhance the interaction of rituximab-opsonized B cells with natural killer cells and other FcγRIIIa-expressing effector cells. Nevertheless, associations between the V allele and clinical response have been inconsistent across autoimmune diseases and treatment settings and may be influenced by treatment regimen, pharmacokinetic exposure, concomitant therapy, disease context, and the depth of B-cell depletion. To our knowledge, no PV-specific association study has established *FCGR3A* rs396991 as a pretreatment predictor of rituximab response. It should therefore be retained only as an exploratory candidate requiring prospective PV-specific evaluation [28,35].

*FCGR2A* rs1801274 is another nonsynonymous variant, producing a histidine-to-arginine substitution at position 131, described as *FCGR2A* H131R or R131H. FCγRIIa is expressed on myeloid effector cells and participates in immune-complex binding and antibody-dependent phagocytosis. The H131 and R131 variants differ in immunoglobulin-binding affinity and could therefore modify the clearance of rituximab-opsonized B cells. However, evidence linking *FCGR2A* rs1801274 to rituximab response is inconsistent, and meta-analytic data from autoimmune diseases have not demonstrated a robust overall association [28].

*IL6* rs1800795 is a functional promoter variant located at -174G>C. Interleukin-6 contributes to B-cell differentiation, plasma-cell survival, inflammatory signaling, and autoantibody production. The CC genotype has been associated with reduced rituximab efficacy in rheumatoid arthritis and in mixed cohorts of systemic autoimmune diseases [41]. This evidence provides a biological rationale for evaluating *IL6* rs1800795 in PV, but no PV-specific predictive validation is currently available.

Common *IL10* promoter variants include rs1800896 (-1082A>G), rs1800871 (-819C>T), and rs1800872 (-592C>A). These variants may influence IL-10 transcription and immunoregulatory activity. IL-10 can support B-cell survival and antibody production while also exerting anti-inflammatory effects, making its relationship with rituximab response biologically complex. Evidence regarding *IL10* variants has primarily been derived from other autoimmune diseases, inflammatory disorders, or responses to non-rituximab biological therapies. Consequently, *IL10* variants should presently be regarded as exploratory immune-regulatory candidates rather than established pharmacogenomic markers of rituximab response in PV [42,43].

*TNF* rs1800629 (-308G>A) is a promoter variant associated with altered *TNF* transcription and inflammatory activity. Although it has been investigated in relation to disease severity and therapeutic response in immune-mediated diseases, available evidence does not establish it as a predictor of rituximab efficacy in PV. Its inclusion in future models would therefore require a prespecified mechanistic hypothesis and independent empirical evaluation [43].

*TNFRSF13B* encodes the transmembrane activator and calcium-modulator and cyclophilin ligand interactor (TACI), a receptor involved in BAFF- and APRIL-mediated B-cell maturation, immunoglobulin class switching, plasma-cell differentiation, and immune homeostasis. Functional variants such as p.Cys104Arg and p.Ala181Glu have been described mainly in common variable immunodeficiency and related immune dysregulation phenotypes. At present, there is insufficient evidence to support a specific *TNFRSF13B* polymorphism as a predictor of rituximab response in PV. *TNFRSF13B* should therefore be presented as a mechanistically plausible research candidate rather than as a selected predictor ready for AI-model implementation [44].

HLA class II alleles require population-specific interpretation because allele frequencies, haplotype structures, and PV-associated effects vary across ancestral and geographical populations. HLA-DRB1*04:02 and HLA-DQB1*05:03 are among the most consistently reported PV susceptibility alleles, but they should not be regarded as universally applicable predictors of rituximab outcomes. Any future model incorporating HLA variables must define its target population and undergo population-specific calibration and external validation before application to other groups. Accordingly, the present conceptual framework has not been validated for any specific ancestral or geographical population, and any future population-specific application will require dedicated model development, calibration, and external validation [45,46].

Importantly, none of these genetic variants has been prospectively validated as a pretreatment predictor of rituximab response in PV. Their inclusion in the proposed framework reflects biological plausibility and evidence derived mainly from other autoimmune diseases. Future PV-specific studies should genotype prespecified variants, define response outcomes prospectively, account for population ancestry and treatment-related covariates, and evaluate whether genetic information adds predictive value beyond clinical, serological, and cellular variables.

### 2.4. Limitations of Individual Biomarkers in Predicting Rituximab Response

Despite their biological relevance, no individual genetic, serological, cellular, clinical, or oral phenotypic biomarker has demonstrated sufficient predictive performance to support independent use in routine clinical practice [11,12,13,28,41,42,43,44,47,48,49]. For example, *FCGR3A* V158F may influence rituximab-mediated antibody-dependent cellular cytotoxicity, but its association with clinical response has not been consistent across diseases and treatment settings [28]. Similarly, changes in anti-DSG3 autoantibody levels may reflect disease activity and relapse risk, although persistent seropositivity can coexist with remission and clinical outcomes cannot be inferred reliably from a single serological measurement [11,12,13].

Comparable limitations apply to BAFF concentrations, CD19^+^ B-cell depletion, and CD27^+^ memory B-cell repopulation, which provide complementary information regarding B-cell depletion and immune reconstitution but have not been validated as independent predictors of rituximab outcome [47,48,49]. Clinical severity indices and oral phenotypic characteristics may further contribute to disease characterization, but their independent predictive value remains insufficiently established [11,12,13,19,20,21]. The principal value of these variables may therefore lie in their task-specific integration within multimodal models rather than in isolated interpretation. This limitation provides the rationale for evaluating artificial intelligence and machine-learning methods capable of combining heterogeneous biological and clinical information in future rituximab outcome models [14,15].

Evidence from other oral inflammatory and resorptive disorders further supports the mechanistic relevance of cytokine networks and host genetic variation, including IL1A, IL1B, and IL1RN polymorphisms; however, these findings are context-specific and should not be extrapolated to rituximab response in PV without dedicated disease- and treatment-specific validation [50,51].

### 2.5. Intended Prediction Tasks and Clinical Use Cases

The proposed framework distinguishes between pretreatment prognosis and post-treatment dynamic monitoring. These tasks involve different index times, eligible predictor sets, outcome definitions, prediction horizons, and potential clinical actions and should therefore not be combined within a single undifferentiated model. In particular, post-treatment pharmacodynamic variables cannot be used in a model intended to generate predictions before rituximab administration.

Pretreatment prognostic task. For baseline prognostic modeling, the index time is defined as the clinical assessment immediately before the first infusion of a rituximab treatment cycle. Only variables available at or before this time are eligible as predictors. Candidate baseline variables may include age, sex, disease duration, previous treatments, baseline systemic corticosteroid exposure, previous relapse history, comorbidities, baseline PDAI, standardized oral disease characteristics, anti-DSG1 and anti-DSG3 antibody levels, baseline peripheral B-cell counts, prespecified genetic variables, and the planned rituximab regimen and dose schedule.

The proposed primary outcome for this task is complete remission off therapy at 12 months after the first rituximab infusion. In accordance with the international consensus definitions for pemphigus, complete remission off therapy is defined as the absence of new or established lesions while the patient has received no systemic therapy for at least 2 months [52]. Complete remission on minimal therapy at 12 months may be evaluated as a secondary outcome and is defined as the absence of new or established lesions while the patient is receiving minimal therapy for at least 2 months [52]. Minimal therapy comprises prednisone or an equivalent corticosteroid dose of no more than 10 mg/day and/or minimal adjuvant therapy.

The intended clinical use of the pretreatment model is prognostic stratification to support patient counseling, planning of follow-up intensity, and prospective research stratification. The model is not intended to determine eligibility for rituximab, justify withholding an effective treatment, select a rituximab dosing regimen, or generate an automatic therapeutic recommendation.

Early-treatment dynamic monitoring task. A separate dynamic model may be defined using a 3-month landmark after the first rituximab infusion. The eligible predictor set may include baseline variables together with information obtained during the interval from baseline to the 3-month landmark, including changes in PDAI and oral disease burden, anti-DSG1 and anti-DSG3 antibody trajectories, cumulative rituximab exposure, systemic corticosteroid exposure, treatment adherence, adverse events, serum rituximab concentration, anti-rituximab antibodies, and the extent of peripheral CD19^+^ B-cell depletion.

For patients who have achieved disease control by the 3-month landmark, the proposed outcome is relapse between months 3 and 12 after rituximab initiation. Relapse is defined according to the international consensus as the appearance of at least three new lesions within 1 month that do not heal spontaneously within 1 week, or the extension of established lesions, in a patient who has previously achieved disease control [52]. The prediction horizon therefore extends from the 3-month landmark to 12 months after treatment initiation. A longer horizon, such as relapse by 24 months, may be evaluated in future studies as a separate outcome and should not be pooled with the 12-month endpoint.

The intended clinical use of the dynamic monitoring model is to identify patients who may benefit from closer clinical and serological surveillance and structured multidisciplinary reassessment. A model-generated estimate should not directly trigger retreatment, treatment escalation, or modification of the rituximab regimen without clinical evaluation.

Inadequate therapeutic response. Inadequate response is not retained as a third, independent prediction task in the present framework because the term has been used inconsistently and may overlap with failure to achieve remission, delayed response, or treatment failure. Where this outcome is investigated in future studies, it must be prespecified using an explicit time point and a consensus-based clinical definition. For example, failure to achieve complete remission on minimal therapy by 12 months could be evaluated as the complementary outcome of the baseline remission model. It should not be combined with relapse, because relapse requires previous achievement of disease control and represents a clinically distinct event.

Each prediction task should therefore be developed and validated separately. Separate analyses are required for baseline remission prognosis and post-treatment relapse monitoring, with task-specific eligibility criteria, predictor time windows, outcome definitions, prediction horizons, and clinical-use statements. The intended tasks and clinical use cases are summarized in Table 2.

### 2.6. Conceptual Architecture and Candidate Data Domains of the Proposed AI Platform

The proposed framework is conceptualized as a task-specific, multilayered architecture for integrating multimodal information relevant to future rituximab outcome modeling in pemphigus vulgaris. Rather than assigning predefined weights or generating clinical recommendations, the framework organizes candidate variables according to their biological domain, temporal availability, and potential role in either pretreatment prognosis or post-treatment response monitoring [14,15,53,54].

The proposed data domains include genetic and pharmacogenomic variables, immunological and serological biomarkers, cellular parameters, clinical characteristics, and oral phenotypic features. Candidate genetic variables include *FCGR3A* rs396991, *FCGR2A* rs1801274, *IL6* rs1800795, and selected exploratory immune-regulatory or population-specific variants, none of which has been validated specifically for rituximab response prediction in PV [28,41,42,43,44,45,46]. Immunological and serological variables include anti-DSG1 and anti-DSG3 autoantibodies, BAFF concentrations, and cytokine profiles, whereas cellular parameters include CD19^+^ B-cell depletion, CD27^+^ memory B-cell repopulation, regulatory T-cell dynamics, and natural killer cell phenotypes [11,13,47,48,49,55]. Clinical and oral phenotypic variables include disease severity, treatment history, previous relapse, comorbidities, oral lesion severity and distribution, desquamative gingivitis, and functional impairment [5,6,19,20,21].

The eligibility of each variable depends on the predefined prediction task and the time at which the information becomes available. Baseline variables may be evaluated within the Pretreatment Prediction Component, whereas variables measured after rituximab initiation are eligible only for the Post-Treatment Response Monitoring Component. The candidate data domains are summarized in Table 3.

**Figure 1:**
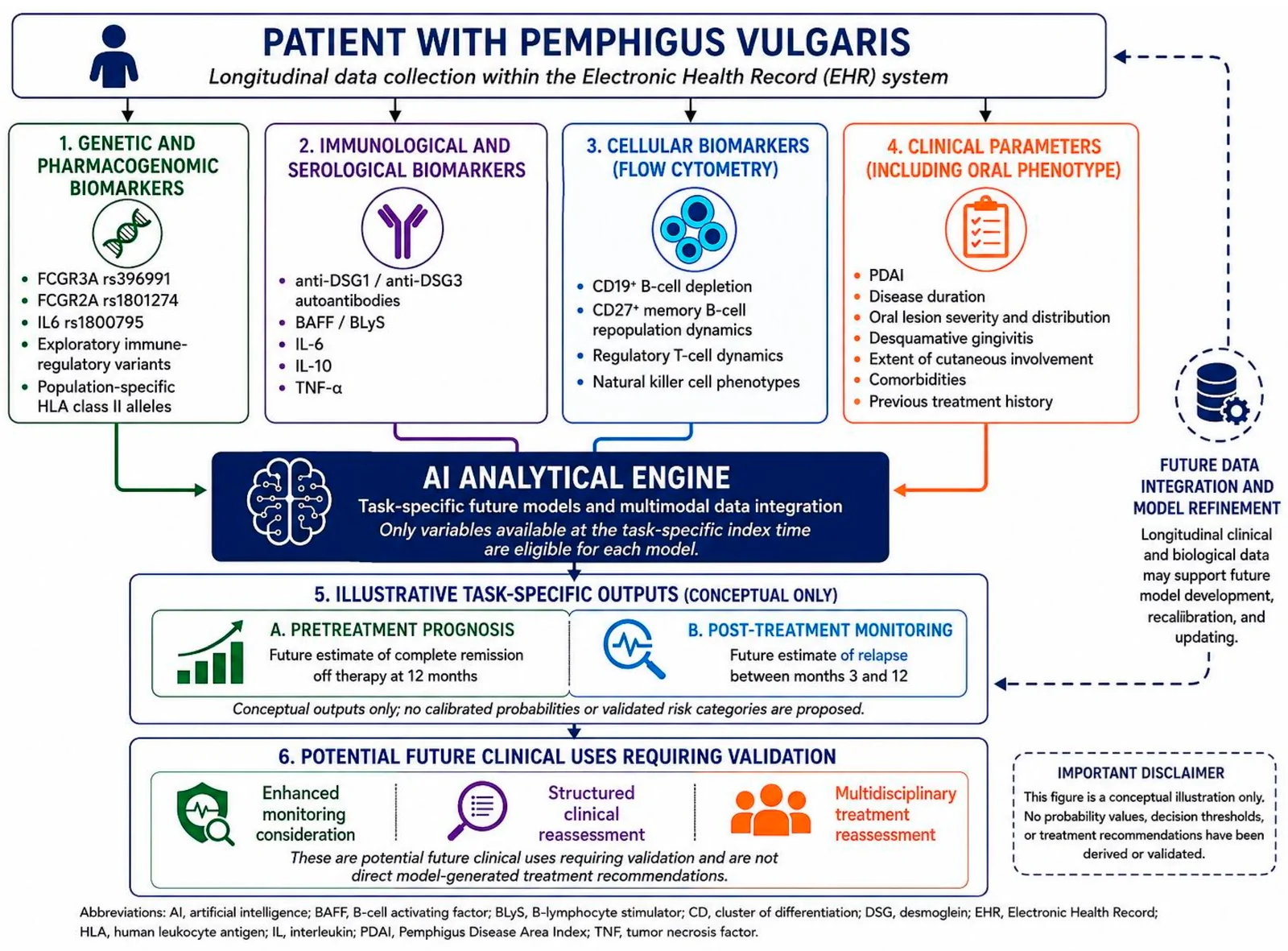
Conceptual architecture of the proposed artificial intelligence-based platform for predicting rituximab response in pemphigus vulgaris. Longitudinal patient information collected within an EHR system is organized into genetic and pharmacogenomic, immunological and serological, cellular, and clinical domains, including the oral phenotype. These data may be integrated within a task-specific artificial intelligence analytical engine to generate an illustrative relative response profile. The potential future clinical uses shown in the figure, including enhanced monitoring, structured reassessment, and multidisciplinary treatment reassessment, would require prospective model development, calibration, external validation, and independent clinical evaluation. The figure is conceptual; no numerical probabilities, decision thresholds, or treatment recommendations have been derived from patient-level data. Abbreviations: AI, artificial intelligence; BAFF, B-cell activating factor; BLyS, B-lymphocyte stimulator; DSG, desmoglein; EHR, Electronic Health Record; HLA, human leukocyte antigen; IL, interleukin; PDAI, Pemphigus Disease Area Index; *TNF*, tumor necrosis factor.

Figure 1 illustrates the conceptual organization of these candidate data domains within a task-specific analytical framework. The eligibility of each variable depends on its temporal availability and on whether it is evaluated within the Pretreatment Prediction Component or the Post-Treatment Response Monitoring Component. Detailed requirements for EHR-based data acquisition, preprocessing, harmonization, model development, validation, and potential updating are presented separately in Section 2.7.

### 2.7. EHR-Based Data Infrastructure and Analytical Workflow

The future development of artificial intelligence-based rituximab outcome models in pemphigus vulgaris requires standardized and longitudinal data-collection infrastructures. Electronic Health Record systems may support the structured acquisition of diagnostic, clinical, oral phenotypic, laboratory, treatment-related, and follow-up information generated throughout the patient’s clinical course [56,57,58].

Within the proposed framework, candidate data domains include demographic and disease-history variables, baseline disease severity, oral lesion characteristics, anti-DSG1 and anti-DSG3 autoantibody levels, circulating cytokines, BAFF concentrations, flow cytometric parameters, and prespecified genetic and pharmacogenomic variables [5,20,28,42,47,48,49]. The integration of these domains into harmonized multicenter registries may increase cohort size, improve population representativeness, and provide the standardized datasets required for future model development and external validation [57,58,59,60,61].

Before analysis, the collected data would require cleaning, standardization, normalization, anonymization, interoperability assessment, and prespecified management of missing values. Separate task-specific analytical datasets should be created for pretreatment prognosis and post-treatment response monitoring. Predictor eligibility must be determined according to the predefined index time, ensuring that variables measured after rituximab initiation are not incorporated into the Pretreatment Prediction Component [62].

Appropriately selected statistical or machine-learning methods may subsequently be evaluated using these harmonized datasets. However, any future model development, updating, or continuous-learning procedure would require internal validation, calibration assessment, external validation, prospective performance monitoring, periodic recalibration, and appropriate governance before clinical implementation could be considered [63,64].

**Figure 2:**
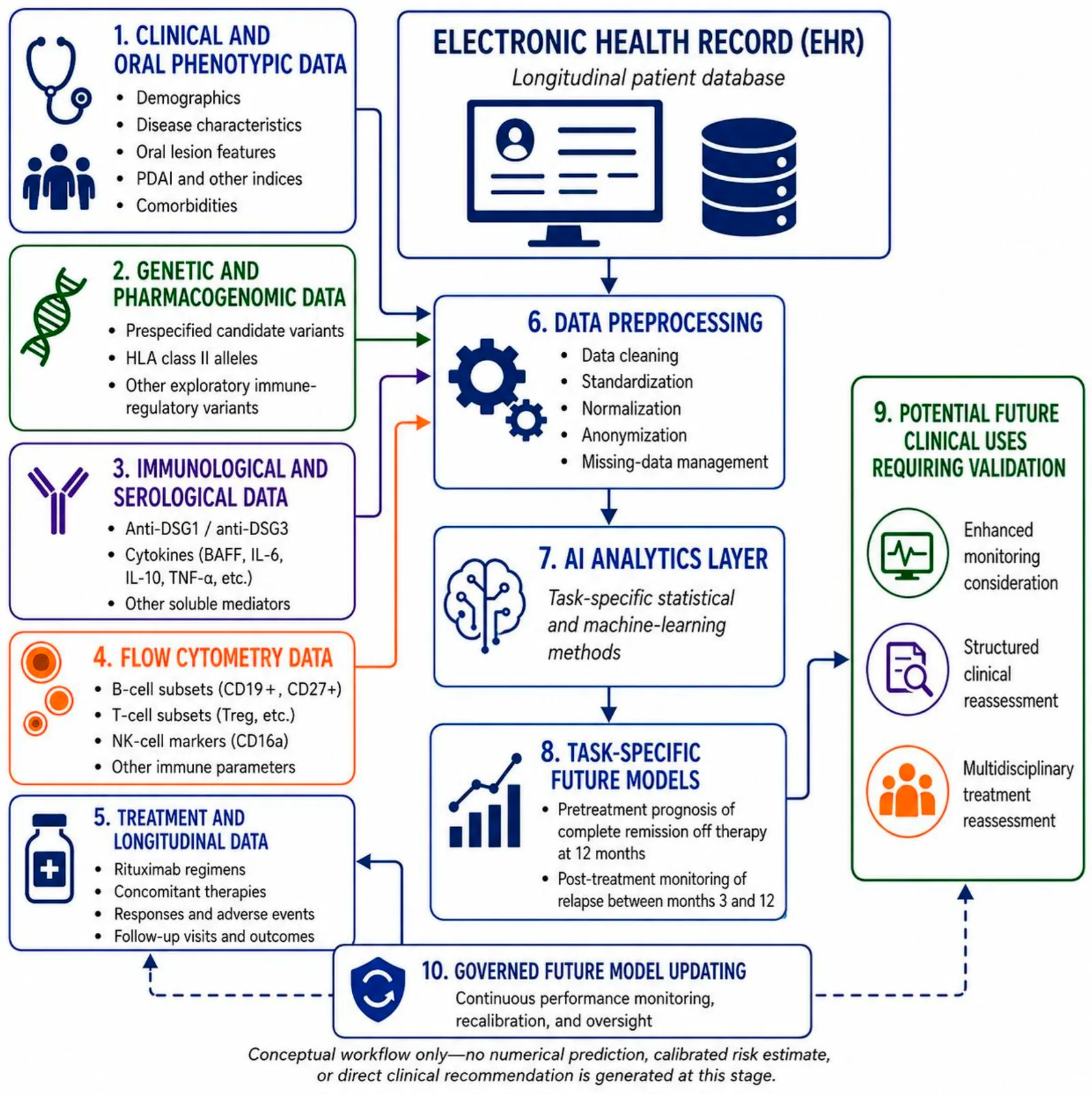
Proposed Electronic Health Record–artificial intelligence workflow for task-specific rituximab outcome modeling in pemphigus vulgaris. Clinical and oral phenotypic data, prespecified genetic and pharmacogenomic variables, immunological and serological biomarkers, flow cytometry findings, and treatment-related longitudinal information are collected within a structured EHR system. Following data cleaning, standardization, normalization, anonymization, and missing-data management, the integrated dataset may be analyzed using appropriately selected and validated statistical or machine-learning methods. Separate future models are proposed for pretreatment prognosis of complete remission off therapy at 12 months and post-treatment monitoring of relapse between months 3 and 12. Any future model updating would require prospective validation, performance monitoring, recalibration, and appropriate governance. The workflow is conceptual and does not generate numerical predictions, calibrated risk estimates, or clinical recommendations at the present stage. Abbreviations: AI, artificial intelligence; BAFF, B-cell activating factor; CD, cluster of differentiation; DSG, desmoglein; EHR, Electronic Health Record; HLA, human leukocyte antigen; IL, interleukin; PDAI, Pemphigus Disease Area Index; *TNF*, tumor necrosis factor.

The proposed EHR–AI analytical workflow is illustrated in Figure 2.

## 3. RTX-AI Response Framework: A Conceptual Architecture for Pretreatment Prediction and Longitudinal Monitoring

### 3.1. Rationale for Developing an Integrative Response Framework

Integrative clinical prediction models may combine multiple patient-level variables to estimate the probability of future outcomes and support individualized decision-making. However, the development of such models requires empirical derivation, assessment of predictive performance, calibration, and validation before numerical outputs or clinical thresholds can be interpreted or applied [56,65].

In PV, treatment response to rituximab remains highly heterogeneous, ranging from prolonged complete remission to early relapse or inadequate therapeutic benefit [11,13]. Although numerous genetic, immunological, serological, cellular, and clinical biomarkers have been associated with treatment outcomes, most have been investigated individually, limiting their predictive utility in routine practice [28,47,49,66]. At present, no validated instrument exists to estimate the individual probability of response to rituximab in patients with PV.

Given the complexity of the biological mechanisms underlying treatment response, there is a growing need for integrative approaches capable of combining information derived from multiple domains into clinically meaningful predictive frameworks. In this context, the development of an integrative response framework may represent a promising strategy for translating multimodal biomarker data into practical decision-support tools aligned with the principles of precision medicine [67].

Accordingly, the RTX-AI Response Framework is presented as a conceptual research architecture illustrating how multimodal candidate variables could be evaluated in future task-specific model-development studies. It should not be interpreted as a validated prediction model, clinical scoring system, or decision-support tool.

### 3.2. Structure of the RTX-AI Response Framework

To illustrate how multimodal biomarkers may be translated into a clinically interpretable framework, we propose a two-stage conceptual model termed the RTX-AI Response Framework. The framework distinguishes between pretreatment response prediction and post-treatment response monitoring according to the time at which the candidate variables become available. This distinction is necessary because baseline predictors and treatment-emergent pharmacodynamic variables address different clinical questions and should not be combined indiscriminately within the same prediction model.

The first stage consists of a Pretreatment Prediction Component designed to integrate candidate variables available before the first administration of rituximab. These variables may derive from genetic, immunological, serological, clinical, and disease-history domains previously associated with rituximab response or disease outcome in PV [28,47,48,49,66]. The second stage consists of a Post-Treatment Response Monitoring Component incorporating pharmacodynamic, serological, cellular, and clinical variables obtained only after treatment initiation.

Unlike approaches based on isolated biomarkers, the RTX-AI Response Framework is intended to capture the combined contribution of multiple determinants of disease activity and therapeutic response. This integrative strategy reflects the principles of precision medicine, according to which clinical outcomes may result from interactions among genetic susceptibility, immune dysregulation, baseline disease burden, previous treatment exposure, and treatment-induced biological changes [53,67].

Pretreatment prediction and post-treatment monitoring represent two distinct analytical tasks. The baseline prediction component aims to estimate the probability of complete remission off therapy at 12 months using only information available at the time of treatment selection. By contrast, the Post-Treatment Response Monitoring Component evaluates the early biological and clinical effects of rituximab after treatment initiation and may be used to update the estimated risk of relapse within the prespecified post-treatment monitoring horizon.

**Figure 3:**
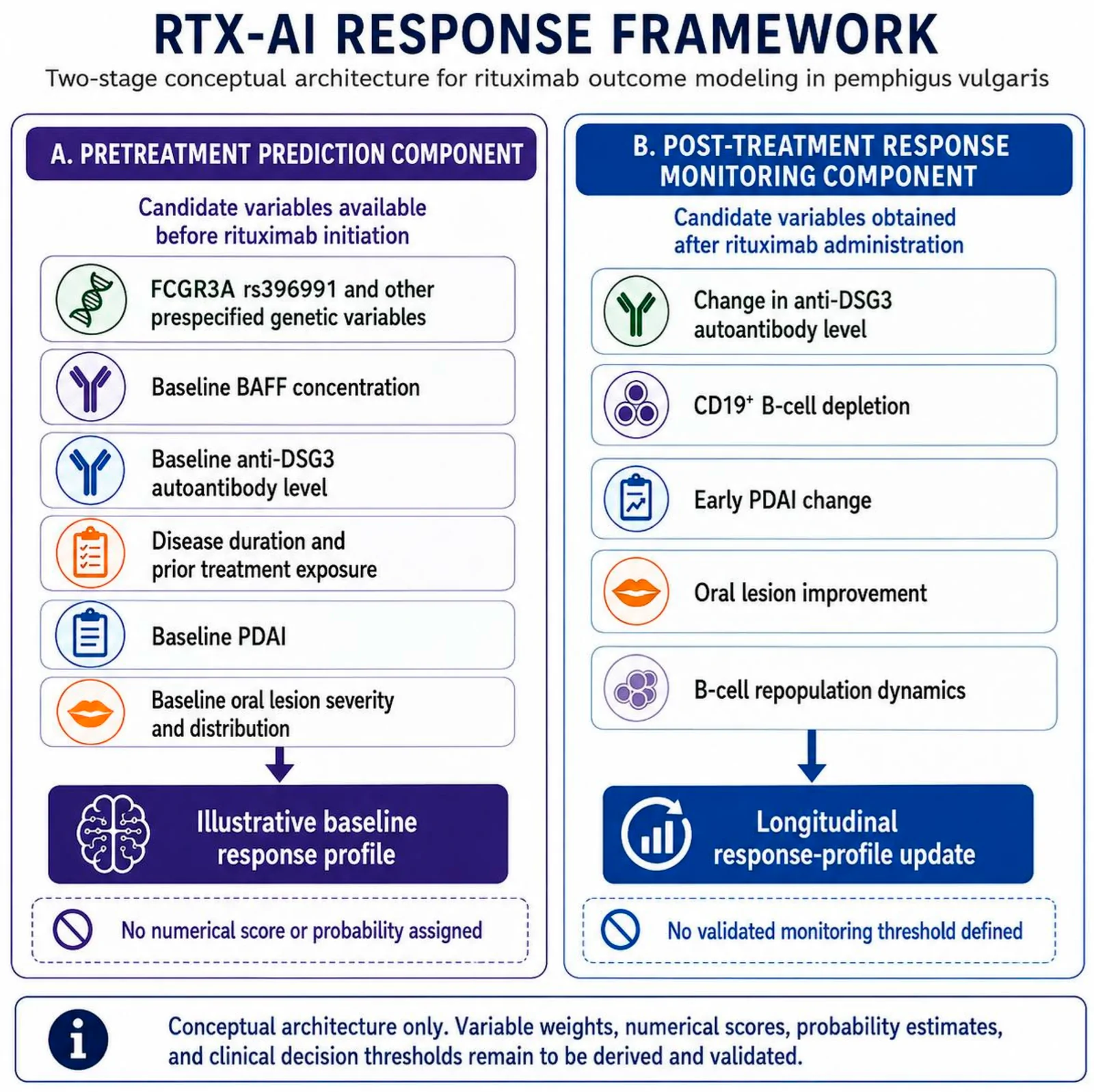
Two-stage RTX-AI Response Framework for pretreatment prediction and post-treatment response monitoring in pemphigus vulgaris. The Pretreatment Prediction Component includes variables available before rituximab initiation, such as prespecified genetic variables, baseline BAFF concentration, baseline anti-DSG3 autoantibody level, disease duration, previous treatment exposure, baseline PDAI, and baseline oral lesion severity and distribution. The Post-Treatment Response Monitoring Component includes treatment-emergent variables, such as changes in anti-DSG3 autoantibody levels, CD19^+^ B-cell depletion, early PDAI changes, oral lesion improvement, and B-cell repopulation dynamics. The temporal separation of these components is intended to prevent target leakage and to distinguish baseline prognosis from longitudinal monitoring. No numerical score, variable weight, probability estimate, monitoring threshold, or clinical decision rule has been derived or validated. Abbreviations: AI, artificial intelligence; BAFF, B-cell activating factor; CD, cluster of differentiation; DSG3, desmoglein 3; PDAI, Pemphigus Disease Area Index; RTX, rituximab.

The conceptual architecture of the revised RTX-AI Response Framework is illustrated in Figure 3. The Pretreatment Prediction Component incorporates six major domains: (i) a genetic component represented by *FCGR3A* polymorphisms; (ii) an immunological component based on baseline BAFF concentrations; (iii) a serological component based on baseline anti-DSG3 autoantibody levels; (iv) a disease-history component incorporating disease duration and previous treatment exposure; (v) a clinical component based on baseline disease severity assessed using the Pemphigus Disease Area Index (PDAI); and (vi) an oral phenotypic component incorporating baseline oral lesion severity and anatomical distribution [5,6,19,20,21,68,69].

Post-treatment variables, including percentage reduction in anti-DSG3 autoantibody levels, the extent of CD19^+^ B-cell depletion, early changes in PDAI, improvement in oral lesions, and subsequent B-cell repopulation dynamics, are not included in the baseline prediction component. These variables become available only after rituximab administration and are therefore incorporated separately within the Post-Treatment Response Monitoring Component. They should be interpreted as indicators of early pharmacodynamic or clinical response rather than as pretreatment predictors [11,13,47,48,49].

This temporal separation is intended to prevent target leakage and preserve the clinical interpretability of the proposed framework. In future model-development studies, the prediction time point should be prespecified, and model inputs should be restricted to variables available at or before that time point. Variables collected after treatment initiation should be evaluated separately using landmark analyses, longitudinal models, or dynamic prediction approaches designed to update response estimates during follow-up [56,70].

An illustrative set of baseline candidate variables for the Pretreatment Prediction Component is presented in Table 4. These variables are included solely to demonstrate the domains that may be considered in future model-development studies and should not be interpreted as components of a clinically validated algorithm. 

By restricting the Pretreatment Prediction Component to variables available before rituximab initiation, the revised framework avoids incorporating early treatment effects into the initial prediction task. The proposed pretreatment component therefore represents a conceptual framework for future model development, whereas treatment-emergent variables are evaluated separately within the longitudinal monitoring component.

The Post-Treatment Response Monitoring Component incorporates variables measured after rituximab administration, including percentage reduction in anti-DSG3 autoantibody levels, complete or partial CD19^+^ B-cell depletion, early changes in PDAI, improvement in oral lesion severity, corticosteroid dose reduction, and subsequent B-cell repopulation. These variables provide information regarding the pharmacodynamic and clinical effects of rituximab and may be evaluated for updating the estimated risk of relapse within the prespecified post-treatment monitoring horizon [11,13,47,48,49].

These post-treatment variables should not be interpreted as pretreatment predictors. Their additional value should be evaluated at prespecified follow-up time points and compared with the performance of the baseline model alone. Future studies should determine whether dynamic integration of early treatment-response variables improves discrimination, calibration, and clinical utility beyond baseline characteristics [56,70].

### 3.3. Future Model Development, Calibration, and Clinical Interpretation

The RTX-AI Response Framework is not intended to define a predefined numerical score or to establish a priori probability categories. At the current stage, no patient-level dataset has been used to estimate regression coefficients, machine-learning feature weights, absolute response probabilities, or clinically meaningful decision thresholds. Accordingly, no numerical score ranges or probability cutoffs are proposed.

Future development of the pretreatment prediction component should follow established principles for clinical prediction-model research. Candidate variables should be selected and evaluated using appropriately sized, prospectively collected patient cohorts, with careful attention to missing data, nonlinear associations, interactions, overfitting, and the temporal availability of predictors. Model performance should be assessed using measures of discrimination and calibration, together with internal validation procedures such as bootstrapping or cross-validation [65,71,72,73].

The development and reporting of future models should follow contemporary guidance for clinical prediction research. TRIPOD+AI should be used to ensure transparent reporting of regression- and machine-learning-based prediction models, whereas PROBAST+AI should inform structured assessment of risk of bias and applicability during model development and external validation. If a model progresses to prospective testing within clinical workflows, early-stage evaluation should be aligned with DECIDE-AI principles, including assessment of human–AI interaction, workflow integration, usability, safety, and unintended consequences. These standards address different stages of translation and should therefore be considered complementary rather than interchangeable [65,74,75].

Because discrimination alone does not establish the reliability of predicted probabilities, calibration should be evaluated using calibration plots, calibration-in-the-large, and calibration slope estimates. External validation in independent multicenter cohorts will subsequently be required to evaluate model transportability and generalizability across different populations, treatment protocols, and clinical settings [71,76].

Any future risk categories or clinical decision thresholds should be derived only after the model has demonstrated adequate predictive performance and calibration. Their potential clinical value should then be evaluated using methods such as decision-curve analysis and should incorporate clinical consequences, patient preferences, treatment-related risks, and expert multidisciplinary judgment. Until these steps have been completed, the RTX-AI Response Framework should be interpreted exclusively as a conceptual architecture for future research and not as a clinical scoring system or decision-support instrument [65,76,77].

### 3.4. Limitations of the RTX-AI Response Framework

The RTX-AI Response Framework remains a conceptual research architecture and has not been developed, trained, calibrated, or validated using patient-level data. Accordingly, the independent and incremental predictive value of the proposed variables, their optimal combination, and the achievable model performance and clinical utility remain unknown. The principal methodological, technological, ethical, and implementation-related limitations relevant to the future development of the framework are discussed comprehensively in Section 5.

## 4. Digital Twin as a Future Research Extension 

A Digital Twin is a dynamic virtual representation of an individual patient that can be updated longitudinally as new biological and clinical information becomes available [78,79,80]. In pemphigus vulgaris, future Digital Twin systems could integrate Electronic Health Record data, genomic and immunological information, serological and cellular biomarkers, oral phenotypic characteristics, treatment-related variables, and longitudinal follow-up data to explore hypothetical disease trajectories and therapeutic scenarios [25,78,79,80,81,82,83,84].

At present, this application remains conceptual. No patient-specific Digital Twin has been developed or validated for pemphigus vulgaris, and the proposed framework does not generate calibrated probabilities, treatment recommendations, or patient-specific therapeutic decisions. Future development would require standardized longitudinal datasets, transparent model-updating procedures, calibration assessment, external validation, and appropriate ethical and data-governance safeguards before any clinical application could be considered.

**Figure 4:**
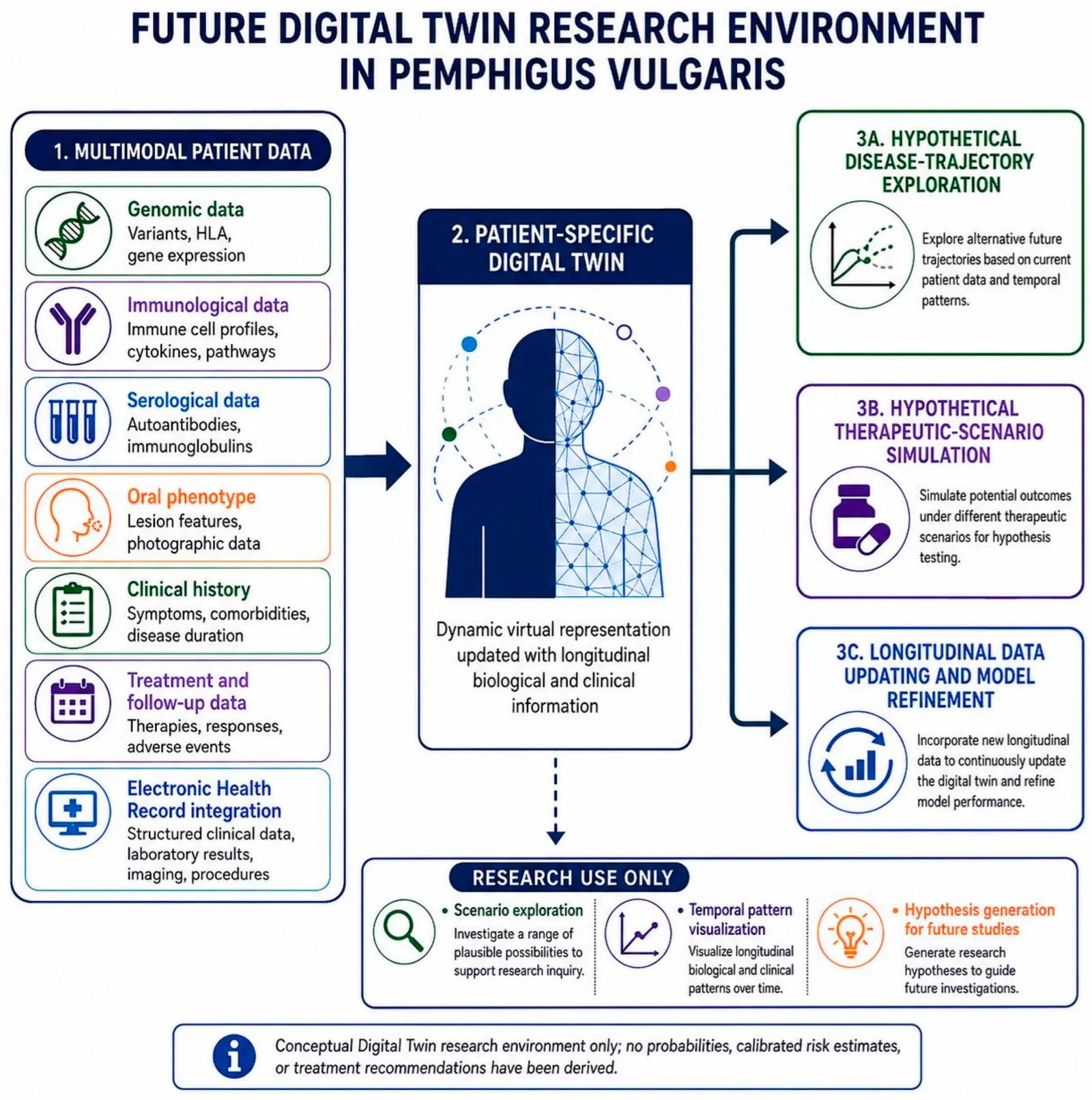
Conceptual future Digital Twin research environment for pemphigus vulgaris. Multimodal patient information, including genomic, immunological, serological, oral phenotypic, clinical, treatment-related, follow-up, and EHR data, may contribute to the future development of a dynamic virtual representation of an individual patient. Such a research environment could support the exploration of hypothetical disease trajectories, therapeutic scenarios, and longitudinal biological and clinical patterns. Continuous incorporation of new data could enable model refinement in future validated systems. The Digital Twin concept presented here is intended exclusively for research and hypothesis generation; no probabilities, calibrated risk estimates, treatment recommendations, or patient-specific therapeutic decisions have been derived. Abbreviations: EHR, Electronic Health Record; HLA, human leukocyte antigen.

The proposed Digital Twin research environment is illustrated in Figure 4.

## 5. Challenges, Limitations, and Ethical Considerations in AI Implementation

The principal challenges affecting the future development and validation of the RTX-AI Response Framework include limited and heterogeneous patient cohorts, variability in rituximab protocols and biomarker-assessment procedures, incomplete longitudinal data, limited EHR interoperability, risks of model overfitting and bias, interpretability requirements, and ethical, data-protection, and regulatory considerations. These issues are addressed in the following subsections.

### 5.1. Limited Patient Cohort Sizes

One of the major challenges associated with the development of artificial intelligence-based predictive models in PV relates to the rarity of the disease. Compared with common chronic conditions such as rheumatoid arthritis, diabetes mellitus, or cardiovascular disorders, the number of patients eligible for prospective studies remains substantially lower [85,86].

This limitation directly affects the performance of machine learning algorithms, which generally require large and diverse datasets for robust training and validation [71,72,85,86,87]. Furthermore, the marked clinical heterogeneity of PV contributes to the additional fragmentation of available cohorts, potentially limiting the ability of predictive models to identify reproducible patterns associated with therapeutic outcomes.

Consequently, the development and validation of reliable AI-driven predictive platforms will require multicenter and international collaborations, together with the harmonization of procedures for the collection, storage, and analysis of biological and clinical data [88,89,90].

Beyond cohort size alone, the choice of analytical approach should be explicitly justified in relation to the expected characteristics of PV datasets. Random Forest and XGBoost are ensemble tree-based methods capable of modeling nonlinear relationships and variable interactions and may be feasible in modest-sized datasets when model complexity is carefully constrained. Missing data should be handled using prespecified methods appropriate to the selected algorithm. Both approaches remain vulnerable to overfitting in small, high-dimensional cohorts and should be compared with simpler penalized regression models using nested cross-validation, calibration assessment, and optimism correction [73].

Outcome class imbalance should be evaluated separately for each task. Discrimination metrics should not be interpreted in isolation when one outcome is uncommon; sensitivity, specificity, positive and negative predictive values, precision–recall curves, and calibration across clinically relevant risk ranges should also be reported. Resampling or class-weighting strategies may be explored within the training data, but they must be applied exclusively within resampling folds to avoid information leakage and should not replace evaluation in cohorts reflecting the natural outcome prevalence.

By contrast, artificial neural networks typically require substantially larger training datasets to achieve stable and generalizable performance and are therefore considered a longer-term objective rather than an immediate modeling strategy. Their inclusion in the proposed framework reflects an anticipated future stage, contingent on the availability of pooled multicenter or federated datasets of sufficient size, consistent with broader methodological strategies proposed for machine learning in small, heterogeneous rare-disease cohorts, including data augmentation and synthetic data generation approaches [91].

Given the longitudinal nature of the candidate variables described in this framework, future model development should also consider methods explicitly designed for repeated-measures or time-to-event data, such as landmark modeling or dynamic prediction approaches, rather than applying standard cross-sectional machine learning algorithms to longitudinal observations without adaptation [70]. Until such comparative evaluations are performed in PV-specific cohorts, the algorithms named throughout this manuscript should be regarded as candidate methods rather than as a validated analytical pipeline.

Sample-size planning should be performed separately for each prediction task and should not rely exclusively on a fixed events-per-variable rule. For illustration, a model evaluating approximately 15 candidate predictor parameters would require substantially more than a single-center cohort of a few dozen patients. Even under a simplified heuristic of 15–20 outcome events per candidate parameter, approximately 225–300 outcome events would be required. If complete remission off therapy at 12 months occurred in approximately 40–60% of participants, the corresponding development cohort could therefore require roughly 375–750 patients before accounting for loss to follow-up, missing data, nonlinear terms, interaction effects, and the need for separate external validation. These figures are illustrative rather than prescriptive; the final sample size should be calculated using prediction-model-specific methods based on the anticipated outcome proportion, number of predictor parameters, expected model performance, and acceptable degree of overfitting [92].

### 5.2. Biomarker Standardization

Another important challenge concerns the lack of methodological standardization in biomarker assessment across different institutions. Variability may arise from the enzyme-linked immunosorbent assay (ELISA) techniques used to quantify anti-DSG1 and anti-DSG3 autoantibodies, the analytical platforms employed for BAFF measurement, differences in flow cytometry protocols, or the genotyping and sequencing technologies adopted by individual research centers [47,48,56,58].

Such methodological discrepancies may introduce systematic bias, reduce the comparability of results, and ultimately influence the predictive performance of artificial intelligence algorithms. Therefore, the development of internationally standardized protocols for biomarker assessment represents a prerequisite for improving reproducibility, external validity, and the clinical applicability of future predictive models [56,58].

### 5.3. Explainability of Artificial Intelligence Models

Many artificial intelligence algorithms operate as “black-box” systems, generating predictions without providing transparent explanations regarding the factors underlying their outputs. In clinical settings, this lack of interpretability may limit physician confidence and represent a significant barrier to the adoption of AI-driven decision-support tools [93,94].

For this reason, the development of Explainable Artificial Intelligence (XAI) has emerged as a priority in biomedical research. Unlike conventional black-box approaches, XAI methodologies enable the identification and quantification of the contribution of individual variables to a given prediction. Techniques such as Shapley Additive Explanations (SHAP) can illustrate how specific biomarkers influence the probability of treatment response, thereby increasing the transparency of algorithmic decisions [94,95].

**Figure 5:**
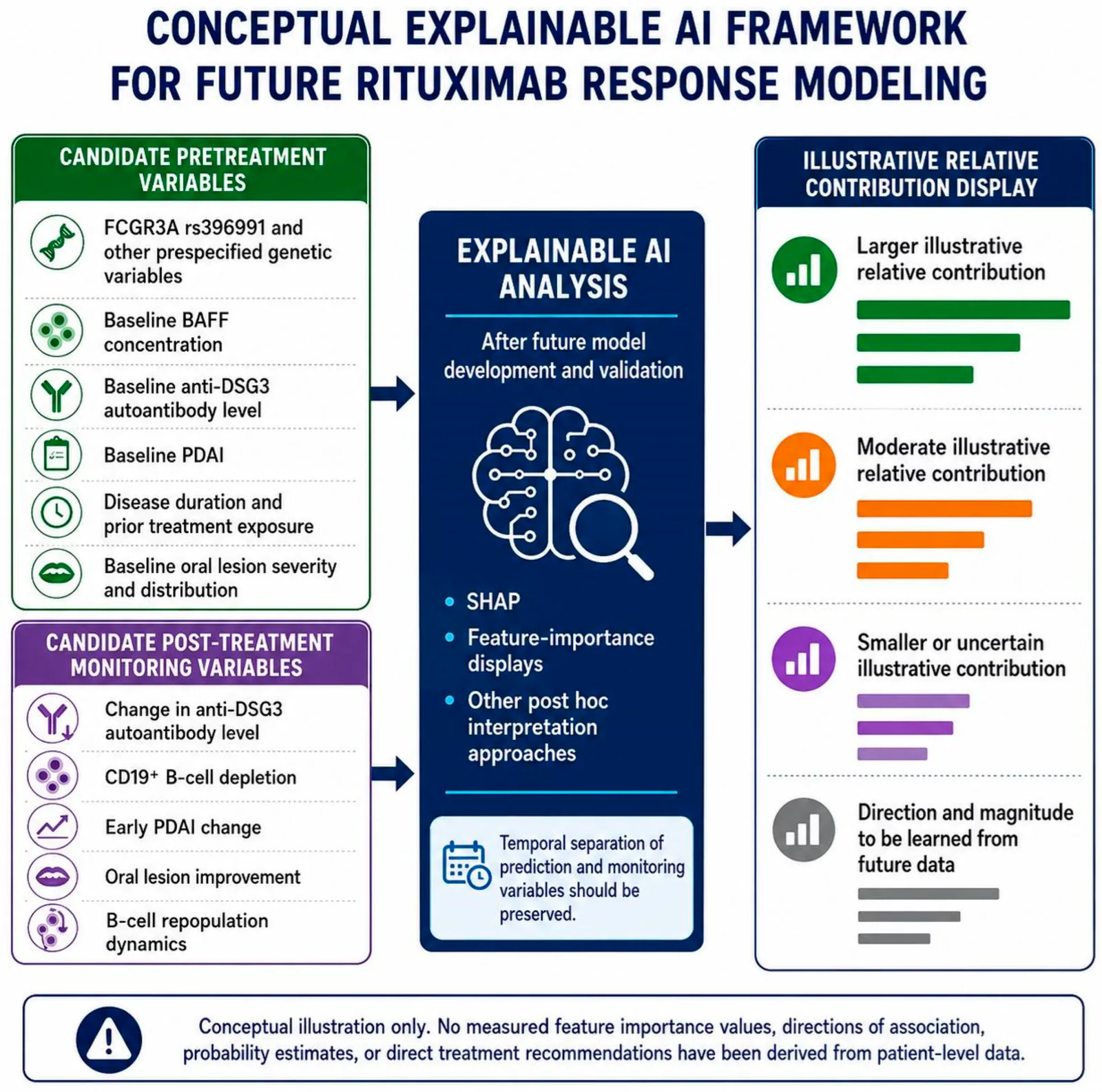
Conceptual illustration of explainable artificial intelligence for future rituximab response modeling in pemphigus vulgaris. Candidate pretreatment variables and post-treatment monitoring variables are shown separately to preserve temporal validity and prevent target leakage. After future model development and validation, interpretation methods such as Shapley Additive Explanations, feature-importance displays, or other post hoc approaches could be used to visualize the relative contribution of individual variables to a model-generated prediction. The bars and contribution categories are schematic and do not represent measured feature importance, established directions of association, numerical effect estimates, probability values, or validated clinical recommendations. Abbreviations: AI, artificial intelligence; BAFF, B-cell activating factor; CD, cluster of differentiation; DSG3, desmoglein 3; PDAI, Pemphigus Disease Area Index; SHAP, Shapley Additive Explanations; XAI, explainable artificial intelligence.

As illustrated in Figure 5, XAI approaches may facilitate the interpretation of complex predictive models by highlighting the relative impact of genetic, immunological, cellular, and clinical variables on the estimated probability of remission. After future model development and validation, XAI methods could be used to identify the variables contributing most strongly to individual model outputs and to support clinically interpretable, independently reviewed assessments [93,94,96].

### 5.4. Ethical Considerations and Data Protection

Artificial intelligence platforms rely on large volumes of sensitive medical information, including genetic data, laboratory findings, clinical records, and treatment histories. Consequently, ensuring data privacy and information security should constitute a fundamental priority in the development and implementation of AI-driven healthcare systems [97,98].

Any platform intended for clinical use must comply with the requirements established by the General Data Protection Regulation (GDPR), the ethical principles governing biomedical research, and internationally recognized standards for the secure management of healthcare data. Data anonymization, database encryption, and rigorous access control procedures represent essential safeguards for the responsible use of artificial intelligence in medical practice [97,99].

Equally important is the need to address issues related to algorithmic fairness, transparency, and accountability. Predictive models trained on unrepresentative datasets may inadvertently perpetuate existing biases or generate inequitable recommendations affecting specific patient populations. Accordingly, efforts aimed at minimizing algorithmic bias and ensuring equitable model performance across diverse populations should be incorporated into the design and validation of future AI systems [100,101].

Despite these challenges, the rapid evolution of multi-omics technologies, the expansion of digital registries, and ongoing advances in artificial intelligence continue to create promising opportunities for the implementation of precision medicine strategies in PV.

## 6. Future Research and Translational Roadmap

Future research should prioritize the establishment of standardized multicenter cohorts and harmonized EHR-based data infrastructures for the prospective evaluation of prespecified clinical, oral phenotypic, serological, cellular, and genetic variables. Given the rarity of pemphigus vulgaris, national and international collaboration will be essential to obtain sufficiently large, diverse, and longitudinal datasets for model development and external validation [56,57,58,59,71,72,85,86,89]. Future extensions may also evaluate genomic, transcriptomic, epigenomic, proteomic, and microbiomic variables when adequate cohort sizes, standardized analytical procedures, and longitudinal data become available [53,54,102,103,104].

Evidence from related clinical domains provides methodological precedents for multimodal biomarker integration, machine-learning-based clinical decision support, and prediction of treatment response during biological therapy [103,104,105,106,107,108,109,110]. Regulatory approaches to biomarker-guided patient selection and statistical methods for identifying treatment-effect heterogeneity may further inform future model specification and evaluation [111,112].

Model development should follow a task-specific sequence that preserves the distinction between pretreatment prognosis and post-treatment response monitoring. The required stages include candidate-variable appraisal, harmonized data collection, explicit definition of target populations, predictor windows, outcomes, and prediction horizons, comparative model development, internal validation and calibration assessment, external validation, evaluation of clinical utility, and prospective assessment of potential implementation under appropriate ethical and data-governance procedures [14,15,58,59,60]. These proposed stages are summarized in Table 5.

**Figure 6:**
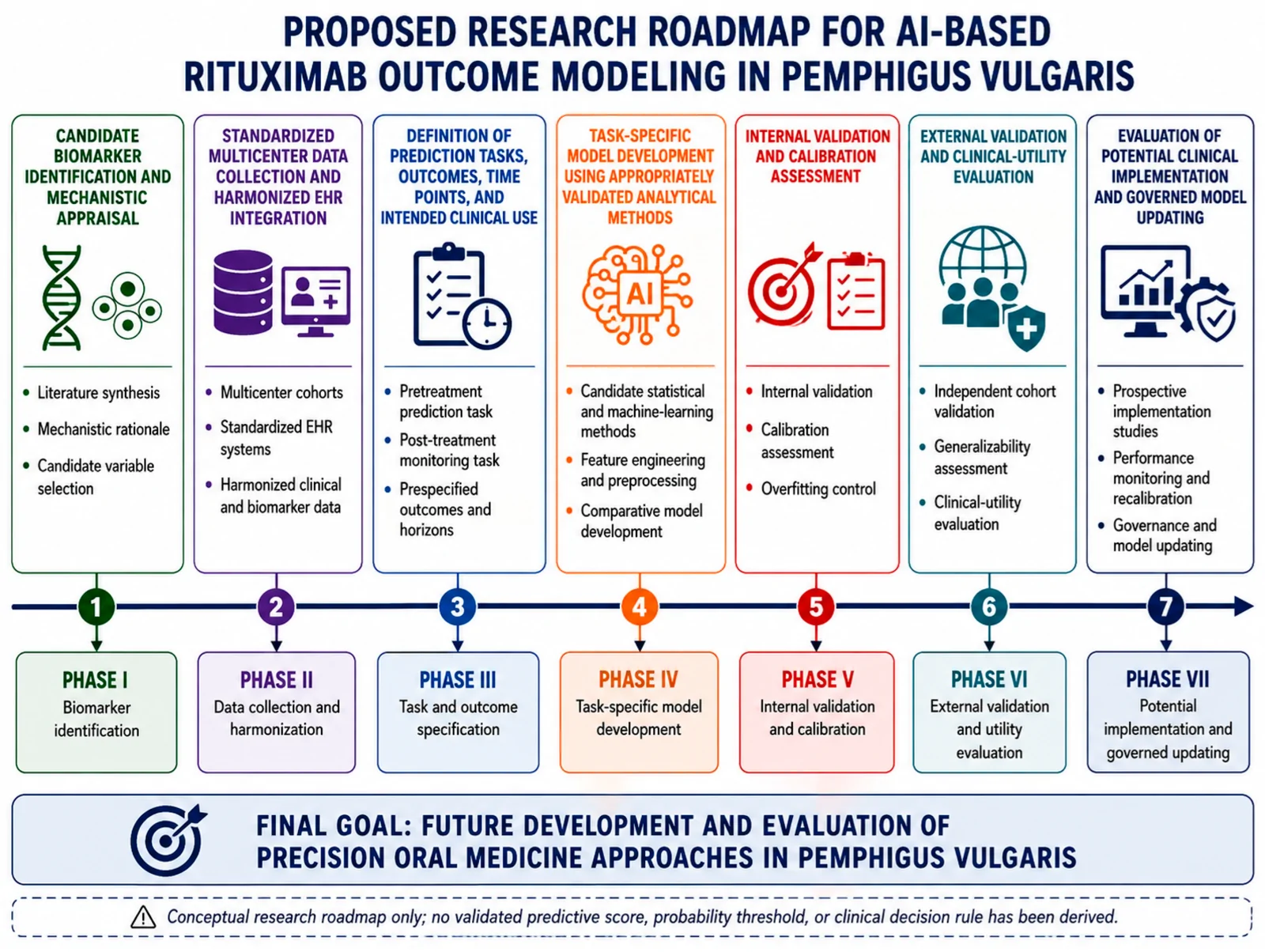
Proposed research roadmap for the development and evaluation of AI-based rituximab outcome models in pemphigus vulgaris. The roadmap includes candidate biomarker identification and mechanistic appraisal, standardized multicenter data collection and harmonized EHR integration, definition of prediction tasks and outcomes, task-specific model development, internal validation and calibration assessment, external validation and evaluation of clinical utility, and subsequent assessment of potential clinical implementation. Any future model updating should be governed by continuous performance monitoring, recalibration, ethical oversight, and data-protection requirements. Clinical implementation should be considered only after adequate predictive performance, calibration, generalizability, and evidence of clinical benefit have been demonstrated. Abbreviations: AI, artificial intelligence; EHR, Electronic Health Record.

A schematic representation of this proposed research pathway is presented in Figure 6.

## 7. Conclusions

Pemphigus vulgaris represents a clinically relevant setting for investigating precision oral medicine approaches to rituximab therapy. Although rituximab has substantially improved therapeutic outcomes, considerable heterogeneity in treatment response remains an important clinical challenge, while oral manifestations contribute substantially to disease burden and longitudinal assessment. Candidate pharmacogenomic, immunological, serological, cellular, clinical, and oral phenotypic variables may provide complementary information; however, none currently supports reliable individual-level prediction when evaluated in isolation.

The proposed RTX-AI Response Framework organizes these variables within two distinct research components: the Pretreatment Prediction Component and the Post-Treatment Response Monitoring Component. It should be interpreted exclusively as a conceptual research architecture rather than as a validated prediction model, clinical scoring system, or decision-support tool. No numerical weights, probability thresholds, or treatment recommendations have been derived from patient-level data.

Future research should therefore focus on prospective multicenter data collection, standardized biomarker assessment, harmonized outcome definitions, task-specific model development, rigorous internal validation, calibration assessment, external validation, and evaluation of clinical utility. Only after adequate predictive performance, calibration, generalizability, safety, fairness, and clinical benefit have been demonstrated should potential clinical implementation be considered.

## Figures and Tables

**Table 1:** Candidate pharmacogenomic variants, biological rationale, and current evidence relevant to future rituximab response modeling in pemphigus vulgaris.

Gene	Variant(s)	Variant Type	Biological Relationship to Rituximab	Current Evidence and Proposed Status
*FCGR3A*	rs396991, p.Phe158Val (F158V/V158F)	Nonsynonymous coding SNV	Alters FcγRIIIa affinity for IgG1 and may influence antibody-dependent cellular cytotoxicity against rituximab-opsonized B cells	Investigated in autoimmune diseases and lymphoma; biologically plausible, but not validated specifically in PV
*FCGR2A*	rs1801274, p.His131Arg (H131R/R131H)	Nonsynonymous coding SNV	Alters FcγRIIa-mediated IgG binding and antibody-dependent phagocytosis	Evidence for rituximab response is inconsistent; exploratory candidate for PV
*IL6*	rs1800795, -174G>C	Promoter SNV	May alter IL-6 expression, B-cell differentiation, plasma-cell survival, and inflammatory activity	Associated with rituximab response in rheumatoid arthritis and mixed systemic autoimmune cohorts; not validated in PV
*IL10*	rs1800896, -1082A>G; rs1800871, -819C>T; rs1800872, -592C>A	Promoter SNVs	May modify IL-10 transcription, immune regulation, B-cell survival, and antibody production	Indirect evidence from other immune-mediated diseases and biological therapies; exploratory only
*TNF*	rs1800629, -308G>A	Promoter SNV	May influence *TNF* transcription and baseline inflammatory activity	No adequate PV-specific or rituximab-specific validation; hypothesis-generating candidate
*TNFRSF13B*	p.Cys104Arg and p.Ala181Glu; other variants require prespecification	Predominantly missense variants	TACI regulates BAFF/APRIL signaling, B-cell maturation, class switching, and immune homeostasis	Mechanistic relevance to B-cell biology, but insufficient evidence for prediction of rituximab response; should not be treated as an established model input
HLA class II	HLA-DRB1*04:02; HLA-DQB1*05:03; other population-specific alleles	Allelic variation	May influence antigen presentation, autoantibody formation, and PV susceptibility	Strong disease-susceptibility evidence, with substantial population- and ancestry-dependent heterogeneity; no established rituximab pharmacogenomic prediction; proposed as population-specific disease-stratification covariates requiring external validation across ancestral groups

Note: The listed variants are candidate variables for future research and are not validated predictors of rituximab response in pemphigus vulgaris. The available evidence is derived predominantly from mechanistic studies, other autoimmune diseases, or other therapeutic settings. Associations reported in other rituximab-treated autoimmune diseases should not be interpreted as validated predictors in pemphigus vulgaris. HLA and cytokine-related variants are included primarily as disease-susceptibility, immune-regulatory, or hypothesis-generating variables. The table does not define a validated pharmacogenomic panel or establish the reliability of a future prediction model. Abbreviations: ADCC, antibody-dependent cellular cytotoxicity; FcγR, Fc gamma receptor; HLA, human leukocyte antigen; IL, interleukin; PV, pemphigus vulgaris; SNV, single-nucleotide variant; TACI, transmembrane activator and CAML interactor; *TNF*, tumor necrosis factor.

**Table 2:** Proposed prediction tasks and clinical use cases for future rituximab outcome models in pemphigus vulgaris.

Component	Pretreatment Prediction Component	Post-Treatment Response Monitoring Component
Target population	Patients with PV scheduled to begin a rituximab treatment cycle	Patients reassessed 3 months after rituximab initiation; relapse analysis restricted to patients who achieved disease control
Index time	Immediately before the first rituximab infusion	Three months after the first rituximab infusion
Predictor window	Information available at or before the first infusion	Baseline information plus data obtained from baseline to month 3
Candidate predictors	Demographic and clinical characteristics; disease duration; treatment history; baseline corticosteroid exposure; PDAI; oral disease characteristics; baseline anti-DSG1/anti-DSG3; baseline B-cell counts; prespecified genetic variables; planned rituximab regimen and dose schedule	Baseline predictors plus changes in PDAI, oral disease burden, anti-DSG1/anti-DSG3 levels, cumulative rituximab exposure, corticosteroid exposure, adverse events, serum rituximab concentration, anti-rituximab antibodies, and CD19^+^ B-cell depletion through month 3
Primary outcome	Complete remission off therapy at 12 months	Relapse occurring after the 3-month landmark and by month 12
Secondary outcome	Complete remission on minimal therapy at 12 months	Relapse by a separately prespecified longer horizon, such as month 24
Prediction horizon	From baseline to 12 months	From month 3 to month 12
Intended clinical use	Prognostic counseling, research stratification, and planning of follow-up intensity	Identification of patients requiring closer surveillance and structured therapeutic reassessment
Actions not supported	Withholding rituximab, selecting dose, or automatically changing treatment	Automatic retreatment, dose escalation, or treatment modification
Post-treatment variables permitted?	No	Yes, but only variables measured on or before the 3-month landmark
Required validation	Internal validation, calibration assessment, and external validation	Landmark-specific internal validation, calibration assessment, and external validation

Note: The Pretreatment Prediction Component and the Post-Treatment Response Monitoring Component represent distinct analytical tasks and should be developed and validated separately. Complete remission and relapse should be defined according to the international consensus definitions for pemphigus. The proposed index times, predictor windows, outcomes, and prediction horizons are research specifications for future model development and do not represent validated clinical decision thresholds. Variables obtained after rituximab initiation are not eligible for inclusion in the Pretreatment Prediction Component. Model-generated estimates should not directly determine rituximab eligibility, dosing, retreatment, treatment escalation, or treatment modification without independent clinical evaluation. Abbreviations: DSG, desmoglein; PDAI, Pemphigus Disease Area Index; PV, pemphigus vulgaris.

**Table 3:** Candidate data domains for future AI-based rituximab outcome modeling in pemphigus vulgaris.

Domain	Examples	Potential Clinical Relevance
Genetic/pharmacogenomic biomarkers	Representative candidate variables summarized in Table 1: *FCGR3A*, *FCGR2A*, *IL10*, *IL6*, *TNF*, *TNFRSF13B*, and population-specific HLA variables	Mechanistic and hypothesis-generating information for future task-specific model development
Immunological and serological biomarkers	Anti-DSG1, anti-DSG3, BAFF, IL-6, IL-10, *TNF*-α	Baseline disease characterization and candidate longitudinal monitoring
Cellular biomarkers	CD19^+^ B-cell depletion, CD27^+^ memory B-cell repopulation, Treg dynamics, NK-cell phenotypes	Baseline immune characterization and post-treatment monitoring, depending on measurement time
Pharmacological and treatment-exposure variables	Rituximab induction regimen, cumulative dose, retreatment schedule, concomitant corticosteroid exposure, serum rituximab concentration, and anti-rituximab antibodies	Adjustment for treatment exposure and candidate post-treatment assessment of pharmacokinetic or immunogenic causes of incomplete depletion and secondary non-response
Clinical variables	PDAI, disease duration, previous treatments, relapse history, corticosteroid exposure, comorbidities	Baseline prognostic characterization and longitudinal outcome assessment
Oral phenotypic variables	Oral lesion severity, lesion distribution, desquamative gingivitis, functional impairment	Baseline prognostic characterization and longitudinal outcome assessment

Note: The listed domains include both candidate baseline predictors and post-treatment monitoring variables. Eligibility for inclusion in a future model depends on the predefined prediction task and the time at which each variable becomes available. Variables obtained after rituximab initiation must not be incorporated into the Pretreatment Prediction Component. In particular, serum rituximab concentration and anti-rituximab antibody status are exclusively post-treatment pharmacological variables and are therefore eligible only for the Post-Treatment Response Monitoring Component. Inclusion in this table reflects potential biological or clinical relevance and does not imply established predictive validity, numerical weighting, or readiness for clinical implementation. Candidate pharmacogenomic variants are detailed in Table 1. Abbreviations: AI, artificial intelligence; BAFF, B-cell activating factor; DSG, desmoglein; HLA, human leukocyte antigen; IL, interleukin; NK, natural killer; PDAI, Pemphigus Disease Area Index; PV, pemphigus vulgaris; *TNF*, tumor necrosis factor; Treg, regulatory T cell.

**Table 4:** Illustrative baseline candidate variables for the Pretreatment Prediction Component of the RTX-AI Response Framework in pemphigus vulgaris.

Domain	Baseline Candidate Variable	Potential Conceptual Contribution
Genetic	*FCGR3A* rs396991 genotype and other prespecified genetic variables	Candidate pharmacogenomic information potentially related to rituximab-mediated effector mechanisms
Immunological	Baseline BAFF concentration	Candidate marker of baseline B-cell survival signaling and immune activity
Serological	Baseline anti-DSG3 autoantibody level	Candidate indicator of baseline serological disease activity
Disease history	Disease duration	Candidate indicator of disease chronicity
Disease history	Previous treatment exposure	Candidate indicator of prior therapeutic burden and treatment refractoriness
Clinical	Baseline PDAI	Candidate measure of baseline disease severity
Oral phenotype	Baseline oral lesion severity and anatomical distribution	Candidate measure of oral disease burden and functional involvement

Note: The variables listed are presented solely as candidates for future model development and are restricted to information available before rituximab initiation. No direction of association, numerical weight, point value, probability estimate, risk category, or clinical decision threshold is proposed. Their independent and combined predictive contributions must be established through prospective model development, internal validation, calibration assessment, and external validation in independent multicenter cohorts. Treatment-emergent variables, including changes in autoantibody levels, B-cell depletion, early PDAI response, and oral lesion improvement, are excluded from the Pretreatment Prediction Component and should be evaluated separately within the Post-Treatment Response Monitoring Component. Abbreviations: BAFF, B-cell activating factor; DSG3, desmoglein 3; PDAI, Pemphigus Disease Area Index; PV, pemphigus vulgaris; RTX, rituximab.

**Table 5:** Proposed research roadmap for the development and evaluation of AI-based rituximab outcome models in pemphigus vulgaris.

Phase	Primary Objective	Expected Outcome
1. Candidate biomarker identification and mechanistic appraisal	Identify candidate genetic, pharmacogenomic, immunological, serological, cellular, clinical, and oral phenotypic variables through literature synthesis and evaluation of their biological relationship to rituximab response.	Prespecified set of biologically plausible candidate variables for future empirical evaluation.
2. Standardized multicenter data collection and harmonized EHR integration	Establish multicenter cohorts and harmonized Electronic Health Record infrastructures using standardized definitions, biomarker-assessment procedures, data dictionaries, and follow-up protocols.	Integrated, interoperable, and quality-controlled longitudinal clinical and biological datasets.
3. Definition of prediction tasks, outcomes, time points, and intended clinical use	Prespecify the target population, index times, eligible predictor windows, outcomes, prediction horizons, and intended clinical use for the Pretreatment Prediction Component and the Post-Treatment Response Monitoring Component.	Clearly defined task-specific research specifications that preserve temporal validity and prevent target leakage.
4. Task-specific model development using appropriately validated analytical methods	Evaluate suitable statistical and machine-learning methods, including data preprocessing, missing-data management, feature engineering, variable selection, and comparative model development.	Candidate task-specific models developed without predefined numerical weights, probability thresholds, or clinical decision rules.
5. Internal validation and calibration assessment	Assess discrimination, calibration, optimism, and overfitting using appropriate internal-validation procedures, such as bootstrapping, cross-validation, or nested cross-validation.	Internally validated and calibrated candidate models with transparently reported performance and uncertainty.
6. External validation and clinical-utility evaluation	Evaluate model transportability, generalizability, calibration, and potential clinical utility in independent multicenter and population-specific cohorts.	Independent evidence regarding predictive performance, calibration, generalizability, fairness, and potential clinical benefit.
7. Evaluation of potential clinical implementation and governed model updating	Conduct prospective implementation studies and establish procedures for performance monitoring, periodic recalibration, ethical oversight, data protection, and protection against model drift.	Evidence-based assessment of potential clinical implementation and a governed framework for future model updating.

Note: The phases shown represent proposed sequential research stages rather than completed model-development, validation, or clinical-implementation steps. The Pretreatment Prediction Component and the Post-Treatment Response Monitoring Component should be developed and validated separately. Potential clinical implementation should be considered only after adequate predictive performance, calibration, generalizability, safety, fairness, and evidence of clinical benefit have been demonstrated. Any subsequent model updating would require prospective governance, continuous performance monitoring, periodic recalibration, and protection against model drift. No numerical score, probability threshold, variable weight, or clinical decision rule has been derived or validated. Abbreviations: AI, artificial intelligence; EHR, Electronic Health Record; PV, pemphigus vulgaris.

## Data Availability

No new data were created or analyzed in this study. Data sharing is not applicable to this article.

## Supplementary Materials

The following supporting information can be downloaded at https://www.mdpi.com/article/10.3390/ph19081292/s1, Supplementary Table S1: Structured database-search strategies and supplementary evidence-identification procedures used for the perspective article.

## Abbreviations

ADCC	Antibody-dependent cellular cytotoxicity
ADCP	Antibody-Dependent Cellular Phagocytosis
AI	Artificial intelligence
APRIL	A Proliferation-Inducing Ligand
BAFF	B-cell activating factor
CD	Cluster of differentiation
DSG1	Desmoglein 1
DSG3	Desmoglein 3
EHR	Electronic Health Record
ELISA	Enzyme-linked immunosorbent assay
*FCGR2A*	Fc gamma receptor IIa
*FCGR3A*	Fc gamma receptor IIIa
FcγR	Fc gamma receptor
GDPR	General Data Protection Regulation
IgG	Immunoglobulin G
HLA	Human leukocyte antigen
IL	Interleukin
ML	Machine learning
NK	Natural killer
PDAI	Pemphigus Disease Area Index
PV	Pemphigus vulgaris
RTX-AI	Rituximab–Artificial Intelligence
SHAP	Shapley Additive Explanations
SNV	Single-Nucleotide Variant
TACI	Transmembrane activator and CAML interactor
Teff	Effector T cells
TNF	Tumor necrosis factor
TNFRSF13B	Tumor necrosis factor receptor superfamily member 13B
Treg	Regulatory T cells
XAI	Explainable Artificial Intelligence
XGBoost	Extreme Gradient Boosting
