# Supplementary material for: Toward Precision Oral Medicine in Pemphigus Vulgaris: A Conceptual AI Framework for Rituximab Response Prediction
Source: Pharmaceuticals (Basel). 2026 Aug 15;19(8):1292. doi: 10.3390/ph19081292 (PMC13516815; doi:10.3390/ph19081292)
Supplement: Supplementary file 1 [file pharmaceuticals-19-01292-s001.zip › pharmaceuticals-4453776-supplementary.pdf]

Supplementary Table S1. Structured database-search strategies and supplementary evidence-identification procedures used for the perspective article

Database/Procedure	Search strategy	Coverage	Last search date
PubMed/MEDLINE	(“pemphigus vulgaris” OR pemphigus) AND (rituximab OR “anti-CD20”) AND (response OR remission OR relapse OR biomarker* OR predictor* OR pharmacogenomic* OR FCGR3A OR FCGR2A OR BAFF OR BLyS OR desmoglein OR “anti-DSG1” OR “anti-DSG3” OR CD19 OR CD27 OR “B-cell depletion” OR “B-cell repopulation”)	Database inception to the last structured search	18 May 2026
PubMed/MEDLINE	(“pemphigus vulgaris” OR pemphigus) AND (“artificial intelligence” OR “machine learning” OR “clinical prediction model” OR “Electronic Health Record” OR “Digital Twin” OR “precision medicine” OR “explainable artificial intelligence”)	Database inception to the last structured search	18 May 2026
Scopus	TITLE-ABS-KEY((“pemphigus vulgaris” OR pemphigus) AND (rituximab OR “anti-CD20”) AND (response OR remission OR relapse OR biomarker* OR predictor* OR pharmacogenomic* OR FCGR3A OR BAFF OR desmoglein OR CD19 OR CD27))	Database inception to the last structured search	18 May 2026
Scopus	TITLE-ABS-KEY((pemphigus OR “autoimmune blistering disease”) AND (“artificial intelligence” OR “machine learning” OR “Electronic Health Record” OR “Digital Twin” OR “precision medicine”))	Database inception to the last structured search	18 May 2026
Web of Science Core Collection	TS=((“pemphigus vulgaris” OR pemphigus) AND (rituximab OR “anti-CD20”) AND (response OR remission OR relapse OR biomarker* OR predictor* OR pharmacogenomic* OR FCGR3A OR BAFF OR desmoglein OR CD19 OR CD27))	Database inception to the last structured search	18 May 2026
Web of Science Core Collection	TS=((pemphigus OR “autoimmune blistering disease”) AND (“artificial intelligence” OR “machine learning” OR “Electronic Health Record” OR “Digital Twin” OR “precision medicine”))	Database inception to the last structured search	18 May 2026
Targeted supplementary	Targeted searches using combinations of the core disease, intervention, biomarker, pharmacogenomic, prediction-model, and	Supplementary evidence identification	5 August 2026

searches and citation checking	artificial-intelligence terms listed above. Reference lists of included publications and relevant reviews were manually screened, and forward citation checking was performed for selected landmark studies and newly published articles relevant to the revised manuscript.	following completion of the structured database searches

Note: Search syntax was adapted to the requirements of each database. The structured database searches were completed on 18 May 2026. Reference lists of included publications and relevant reviews were manually screened, and forward citation searching was performed for selected landmark studies. Targeted supplementary searches and forward citation checking were subsequently conducted through 5 August 2026 to identify newly published studies relevant to the revised manuscript. Because this article was designed as a perspective rather than a systematic review, no PRISMA flow diagram, formal risk-of-bias assessment, or quantitative evidence synthesis was undertaken.
